# Sustainable Recovery and Biofunctional Characterization of Polyphenol-Rich Extracts from Norway Spruce, Chestnut Wood, and Pomegranate By-Products

**DOI:** 10.3390/foods15081422

**Published:** 2026-04-19

**Authors:** Francesca Vidotto, Cristiana Sbrana, Laryssa Peres Fabbri, Andrea Cavallero, Giulia Baini, Luca Tagliavento, Francesco Meneguzzo, Morena Gabriele

**Affiliations:** 1 National Research Council, Institute of Agricultural Biology and Biotechnology (CNR-IBBA), 56124 Pisa, Italy; f.vidotto@student.unisi.it (F.V.); cristiana.sbrana@cnr.it (C.S.); laryssaperesfabbri@cnr.it (L.P.F.); andrea.cavallero@ibba.cnr.it (A.C.); 2 Department of Physical Sciences, Earth and Environment, University of Siena, 53100 Siena, Italy; giulia.baini2@unisi.it; 3 HyRes S.r.l., 82100 Benevento, Italy; luca.tagliavento@hyres.it; 4 National Research Council, Institute of Bioeconomy (CNR-IBE), 50019 Florence, Italy; francesco.meneguzzo@cnr.it

**Keywords:** agri-food and forestry by-products, green extraction technologies, polyphenols, antioxidant activity, antimicrobial activity, enzyme inhibition

## Abstract

In the sustainability framework, valorization of organic by-products as reservoirs of phytochemicals useful for human health represents a hot topic. Therefore, this study evaluated Norway spruce bark and twigs (NSB, NST), chestnut tree wood (CTW), and pomegranate fruit waste/pomace (PFW) as sources of bioactive compounds by employing green technologies. Microwave-assisted extraction (MAE) and ultrasound-assisted extraction (UAE), applied individually or sequentially, were optimized by modulating solvent composition, temperature, time, microwave power, and ultrasound amplitude. Hydroalcoholic extraction (50% ethanol) combined with MAE yielded the highest phenolic recovery and antioxidant activity across all matrices. PFW exhibited the highest antioxidant activity assessed through FRAP, ORAC, and DPPH assays. Phytochemical profiling by HPLC-DAD identified stilbenes in spruce extracts, ellagic acid in chestnut wood, and ellagic acid and punicalagins in pomegranate waste as major bioactive constituents. Additionally, NSB and PFW exhibited α-amylase inhibitory activity. Antimicrobial testing demonstrated dose-dependent activity against Gram-positive (*Staphylococcus epidermidis* and *Bacillus subtilis*) and Gram-negative (*Pseudomonas stutzeri*) strains, with PFW exhibiting the strongest inhibition and NSB displaying broad-spectrum effects. Total phenolic content changed moderately after 21 days of storage. These results demonstrate that sustainable extraction enables efficient recovery of bioactive compounds from plant by-products, supporting their further functional, dietary, and medicinal applications.

## 1. Introduction

The demand for resources from a growing population has many important global consequences [1]. Primarily, this translates into increased industrial production and, consequently, a higher generation of by-products. The burden imposed by waste is huge, representing a loss of energy throughout the production chain, in terms of labor, pollution, and profit, thus causing both economic and environmental damage [2].

In this frame, the concepts of circular economy and sustainability aim at reducing these detrimental externalities by re-integrating by-products into the productive chain, in the form of high-added-value items [3,4]. Of all sectors, the agri-food and forestry business provides huge amounts of organic waste materials, which could help meet the rising need for nutritious human food. Indeed, in the past two decades, scientific studies concerning the valorization of agri-food by-products have surged, demonstrating that, if adequately processed, they can provide components highly desirable to the materials, food, pharmaceutical, and cosmetic industries [5,6].

Plant-based waste products originate both from the food processing sector, such as fruit and vegetable peels, seeds, and pomace, and from the forestry sector, including leaves, flowers, twigs, bark, and wood [7,8].

Extensive research highlights their richness in bioactive and nutritive compounds; among them, proteins, dietary fibers, fatty acids, pigments, and phenolic compounds stand out [9]. Phenolics are specialized metabolites biosynthesized by plants for protective and reproductive purposes, but they are also valuable health-promoting agents, exerting antioxidant, anti-inflammatory, and antimicrobial activities, thereby constituting natural allies against several human pathological conditions [10,11,12].

Nowadays, the recovery of such components is facilitated by the availability of multiple green technologies, which aim at maximizing extraction efficiency while minimizing energy expenditure. This is achieved by leveraging parameters such as solvent type, time, temperature, and pressure. Microwave-assisted extraction (MAE) and ultrasound-assisted extraction (UAE) are amongst the most popular sustainable extraction techniques. The principle of MAE is that microwave energy induces dipole rotation and ionic conduction of solvent molecules, rapidly heating plant cells and disrupting cell walls, thereby enhancing the release of target compounds. Conversely, the UAE relies on high-frequency sound waves (>20 kHz) transmitted through a solvent, generating cavitation bubbles that collapse and disrupt cell structures. Both techniques have been extensively employed for the valorization of organic by-products, as they are straightforward, inexpensive, and effective, consistently reducing extraction time compared to conventional methods [6,13].

Despite the growing number of studies on agri-food by-products, most research has focused on a single matrix or specific extraction technique, with limited comparative analyses across agri-food and forestry residues using scalable green technologies. Furthermore, many investigations are limited to chemical characterization and antioxidant assays, while integrated evaluations including antimicrobial and enzyme-inhibitory activities are less frequently addressed. In this context, the present study aims to systematically evaluate selected agri-food and forestry by-products, including Norway spruce (*Picea abies* L.) bark (NSB) and Norway spruce twigs (NST), chestnut tree (*Castanea sativa* L.) wood (CTW), and pomegranate fruit waste (*Punica granatum* L.) (PFW) products, as sources of bioactive compounds by applying MAE and UAE, either individually or in combination. Particular attention is paid to the influence of extraction parameters on phenolic recovery and biofunctional properties, including antioxidant, antimicrobial, and enzyme-inhibitory activities. By integrating phytochemical profiling and functional assays, this work seeks to provide a comparative assessment of different biomass types within a sustainable valorization framework.

Recent work using hydrodynamic cavitation (HC) showed that these same by-products can yield polyphenol-rich extracts with antimicrobial and/or health-related bioactivities at pilot or semi-pilot scale [14,15,16], suggesting that MAE/UAE results may be positioned within a broader toolbox of scalable green-extraction options.

## 2. Materials and Methods

### 2.1. Reagents

All standards and reagents were of analytical grade. Gallic acid, quercetin, resveratrol, ellagic acid, punicalagins (α + β), ethanol, methanol, acetonitrile, formic acid, Folin–Ciocalteu reagent, AAPH (2,2′-azobis(2-amidinopropane) dihydrochloride), DPPH (2,2-diphenyl-1-picrylhydrazyl), fluorescein, hydrochloric acid, iron(II) sulfate heptahydrate, glacial acetic acid (100%), acetone, sodium chloride, sodium acetate, sodium hydroxide, sodium carbonate, aluminum chloride, potassium chloride, ferric chloride hexahydrate, sodium phosphate, sodium nitrite, TPTZ (2,4,6-tri(2-pyridyl)-1,3,5-triazine), tris(hydroxymethyl)aminomethane hydrochloride, Trolox (6-hydroxy-2,5,7,8-tetramethylchroman-2-carboxylic acid), acarbose, α-amylase type IV-B from porcine pancreas, potato starch, dinitrosalicylic acid, human soluble dipeptidyl peptidase IV, diprotin A (Ile-Pro-Ile), H-Gly-Pro-p-nitroanilide, porcine pancreatic lipase type II, p-nitrophenyl butyrate, orlistat, maltose, MH (Mueller-Hinton), and agar were purchased from Sigma-Aldrich (Saint Louis, MO, USA).

### 2.2. Origin, Nature, and Extraction of Agri-Food and Forestry By-Products

Norway spruce (*Picea abies* (L.) H. Karst.) bark (NSB) and twigs (NST) were collected from mature trees in the Fiemme Valley, eastern Alps, about 1200 to 1800 m a.s.l. (around 46°15′ N, 11°30′ E). No bleaching treatment or dedicated drying step was applied. After collection, the materials were stored under vacuum at −18 °C until use. Before extraction, the samples were milled with a Tritone One Electric shredder (Ceccato Olindo srl, Arsego, Italy) and sieved through an electric vibrating sieve (KXY-062, Yuchengtech, Hangzhou, China) to obtain particles with a size smaller than 1.5 mm.

Pomegranate pomace, consisting of peel and seeds, was supplied by Masseria Fruttirossi Srl, Castellaneta (TA), Italy. The material was transported under frozen conditions at −18 °C within 24 h from collection from the supplier’s refrigerated storage facility. Upon arrival, the by-products were crushed using a fruit mill (model MLP0002, Polsinelli Enologia Srl, Frosinone, Italy), then stored under vacuum at −18 °C until use. The pomace was then stored at −80 °C and subsequently lyophilized using a ScanVac CoolSafe 55-4 lyophilizer (LaboGene ApS, DK-3450 Allerød, Denmark) to obtain pomegranate fruit waste (PFW).

Chestnut wood waste was obtained from a local sawmill (Segheria Tani, Borgo San Lorenzo, Firenze, Italy) and consisted of small leftover wood chips from the production of logs for fire. The material was then sieved (filter mesh of 3 by 3 mm) and stored at room temperature in opaque packaging until use, due to its low water content (<15%).

The plant waste materials, after the matrix-specific pretreatments described above, were extracted in distilled water or 50% (*v*/*v*) ethanol (EtOH) at a solid-to-solvent ratio of 1/30 (*w*/*v*) and homogenized using an Ultraturrax homogenizer (Kinematica Polytron PT MR 2100, Lucerne Switzerland, power: 500 W). For conventional extraction, samples were kept under continuous agitation for 2 h at room temperature. For MAE and UAE, homogenized samples were processed using a UWave-2000 system (Sineo Microwave Chemistry Technology, Shanghai, China), applying variable extraction times and temperatures, microwave power (500 W and 1000 W), or ultrasound amplitudes (50% and 80%), while maintaining continuous agitation. Subsequently, all samples were centrifuged at 2700× *g* for 10 min at 4 °C (Jouan CR31, Newport Pagnell, UK), and the supernatants were collected and stored at 4 °C until analysis. A schematic representation of the extraction workflow and instrumentation is provided in the Supplementary Materials (Figure S1). Extraction conditions evaluated in this study are summarized in Table 1.

### 2.3. Phytochemical Assays

#### 2.3.1. Total Phenolic Content (TPC)

Total polyphenol content was determined using the Folin–Ciocalteu assay, following a previously described method [17] with minor adaptations. Briefly, 100 µL of extracts were mixed with 500 µL of 0.2 N Folin–Ciocalteu reagent and incubated in the dark for 5 min. Subsequently, 400 µL of 0.7 M Na_2_CO_3_ were added, and the mixtures were incubated for 2 h at room temperature in the dark. Absorbance was measured at 760 nm using a microplate reader (FLUOstar Omega Microplate Reader, BMG LABTECH, Ortenberg, Germany). Calibration curves were constructed using gallic acid as a standard in the range of 0.005-0.175 mg/mL, showing good linearity (R^2^ > 0.99). Results were expressed as mg gallic acid equivalents per g of dry weight (mg GAE/g dw).

#### 2.3.2. Total Flavonoid Content (TFC)

Total flavonoid content was assessed using the aluminum chloride colorimetric method, adapted from a previously published protocol [18]. A volume of 100 µL of extract was mixed with 400 µL of H_2_O and 30 µL of 5% NaNO_2_, and the mixture was incubated for 5 min at room temperature. Subsequently, 30 µL of 10% AlCl_3_ was added to each tube, followed, after 6 min, by 200 µL of 1 M NaOH and 240 µL of H_2_O. After a final incubation of 30 min, absorbance was recorded at 430 nm (FLUOstar Omega Microplate Reader, BMG LABTECH, Germany). Calibration curves were constructed using quercetin as the standard over the range of 0.00625–0.250 mg/mL, showing good linearity (R^2^ > 0.99). Results were expressed as mg quercetin equivalents per g of dry weight (mg QE/g dw).

### 2.4. Antioxidant Activity Assays

#### 2.4.1. Ferric Reducing Antioxidant Power (FRAP)

The ferric-reducing capacity of extracts was evaluated using the FRAP assay in accordance with Chelucci et al. [19]. The FRAP reagent was freshly prepared and mixed with extracts at a fixed ratio (735 µL of FRAP reagent + 35 µL extract). After 30 min of incubation in the dark, absorbance was measured at 593 nm (FLUOstar Omega Microplate Reader, BMG LABTECH, Germany). Calibration curves were constructed using FeSO_4_·7H_2_O as the standard over the range of 0.0125–0.560 mg/mL, showing good linearity (R^2^ > 0.99). Results were expressed as mg Fe^2+^ equivalents per g of dry weight (mg Fe^2+^/g dw).

#### 2.4.2. ORAC (Oxygen Radical Absorbance Capacity) Assay

ORAC assay, based on the inhibition of fluorescein oxidation induced by AAPH-derived peroxyl radicals, was carried out as previously reported [20]. Fluorescence decay was monitored at λex 485 nm/λem 514 nm (FLUOstar Omega Microplate Reader, BMG LABTECH, Germany), and results were expressed as µmol Trolox equivalents per g of dry weight (µmol TE/g dw).

#### 2.4.3. DPPH (2,2-Diphenyl-1-picrylhydrazyl) Radical Scavenging Activity

The radical-scavenging activity against the DPPH• radicals was evaluated following a slightly modified protocol [21]. Briefly, 25 µL of extract was mixed with 975 µL of a 60 µM DPPH ethanolic solution. The mixture was kept at 30 °C in the dark for 30 min, and the absorbance was recorded at 515 nm (FLUOstar Omega Microplate Reader, BMG LABTECH, Germany). Results were expressed as EC_50_ (µg/mL).

### 2.5. HPLC-DAD Analysis

Phenolic profiling was performed using an HPLC-DAD (Prominence-i LC-C 3D Plus, N/SL21455510075; Shimadzu Europa GmbH, Duisburg, Germany), following a published protocol [22]. Separation was achieved using water (A) and acetonitrile (B), both containing 0.1% formic acid, at a constant flow rate of 0.8 mL/min. The gradient elution was as follows: 10–25% B over 15 min, increased to 35% at 18 min, and to 50% at 35 min. Absorbance was set at 330 nm for stilbenes (resveratrol used as standard), 254 nm for ellagic acid, and 375 nm for punicalagins (α + β). Retention times and UV–vis spectra were assessed to assign compound identity and classes. Peaks belonging to the same class were integrated and summed. Quantification was performed using calibration curves (R^2^ > 0.99) obtained from certified analytical standards. Results were expressed as mg of standard equivalents per g of dry weight.

### 2.6. Enzymatic Assays

#### 2.6.1. Alpha-Amylase Inhibitory Activity

The α-amylase inhibition assay was adapted from the procedure by Johnson et al. [23]. In summary, 50 μL of extracts, positive control (2 mM acarbose), or negative control (distilled water) were mixed with 100 μL α-amylase type IV-B from porcine pancreas (2 U/mL in 25 mM Tris-HCl, pH 7.5, containing 100 mM KCl). The mixtures were incubated at 20 °C for 5 min at 1000 rpm. Then, 100 μL of a freshly 1% (*w*/*v*) soluble starch solution were added, and the mixtures were incubated for 6 min under the same shaking conditions. The reaction was stopped by adding 100 μL of dinitrosalicylic acid (DNS) reagent, followed by heating at 100 °C for 15 min in a thermoblock. Samples were cooled in an ice bath, and 800 µL of H_2_O was added to each tube. Absorbance was measured at 540 nm using the FLUOstar Omega Microplate Reader (BMG LABTECH, Germany). Results were expressed as a percentage of enzyme activity inhibition, using a standard curve of maltose in the range of 0.100–0.975 mM, showing good linearity (R^2^ > 0.99).

#### 2.6.2. Dipeptidyl Peptidase IV (DPP-IV) Activity

The DPP-IV inhibitory activity was determined according to the method reported by Silveira et al. [24], with minor adaptations. Briefly, 15 μL of recombinant human soluble DPP-IV were incubated for 10 min at 37 °C with extracts in a final volume of 50 μL. Diprotin A and buffer served as the positive and negative controls, respectively. Then, 50 µL of substrate H-Gly-Pro-p-nitroanilide were added to a final concentration of 100 μM. Absorbance was recorded at 405 nm at 37 °C for 30 min at 2 min intervals (FLUOstar Omega Microplate Reader, BMG LABTECH, Germany). The slope of the linear portion of the DPP-IV activity curves with and without samples was used to calculate the percentage inhibition.

#### 2.6.3. Lipase Inhibitory Activity

The inhibition of lipase activity was evaluated by adapting the method of Bustanji et al. [25]. Briefly, 100 µL of porcine pancreatic lipase type II were mixed with 20 µL of extracts, 12.5 µL of p-nitrophenyl butyrate (PNPB), and 867.5 µL of buffer. Orlistat and buffer were used as positive and negative controls, respectively. Absorbance was measured at 410 nm at 1 min intervals for 30 min (FLUOstar Omega Microplate Reader, BMG LABTECH, Germany). The percentage inhibition of lipase was calculated from the slope of the linear portion of the lipase activity curves with and without samples against a denatured enzyme blank.

### 2.7. Antimicrobial Activity

The antimicrobial activity of optimized extracts was evaluated by a broth microdilution susceptibility assay, using *Staphylococcus epidermidis* CNR-MLIP B206, *Bacillus subtilis* CNR-MLIP B038 (food-borne isolates maintained in the culture collection of the CNR-IBBA Microbiology Laboratory, CNR-MLIP), and *Pseudomonas stutzeri* DSM 5190 (clinical isolate from the DSMZ culture collection, maintained at CNR) bacterial strains, as previously outlined by [26]. Extracts were added to bacterial cultures in Mueller–Hinton broth at defined concentrations, maintaining constant the final volume of both extract and cell suspension in each well. Extract concentrations ranged from 0.75 to 24 mg/mL. Bacterial growth was quantified after 24 h at 30 °C by measuring optical density (OD) at 600 nm (FLUOstar Omega Microplate Reader, BMG LABTECH, Germany). Results were expressed as log_10_ cells/mL using reference growth curves.

### 2.8. Statistical Analysis

Data were analyzed using GraphPad Prism v10.0 (GraphPad Software, San Diego, CA, USA). Results are reported as mean values ± standard deviation (SD) or standard error (SE) of at least two independent experiments. Statistical significance was assessed using one-way or two-way analysis of variance (ANOVA), followed by Tukey’s post hoc test, with *p* < 0.05 considered significant. The stability of total phenolic content over 21 days of storage was analyzed by paired Student’s *t*-test.

## 3. Results and Discussion

### 3.1. Optimization of Extraction

To identify the optimal extraction conditions for the investigated waste materials, a series of systematic extraction trials was carried out. The primary objective was to assess whether green extraction techniques, namely MAE and UAE, could provide extraction efficiencies comparable to or superior to conventional maceration in terms of phenolic compound recovery and antioxidant capacity. In addition, the influence of key extraction variables—solvent composition, extraction time, temperature, microwave power, and ultrasound amplitude—was evaluated. All extracts were preliminarily screened for total phenolic content and antioxidant activities, primarily using ORAC and FRAP assays. This optimization strategy was applied to all plant matrices except Norway spruce twigs (NST); due to the close similarity of this material to Norway spruce bark (NSB), the extraction parameters optimized for NSB were directly transferred to NST.

**Figure 1:**
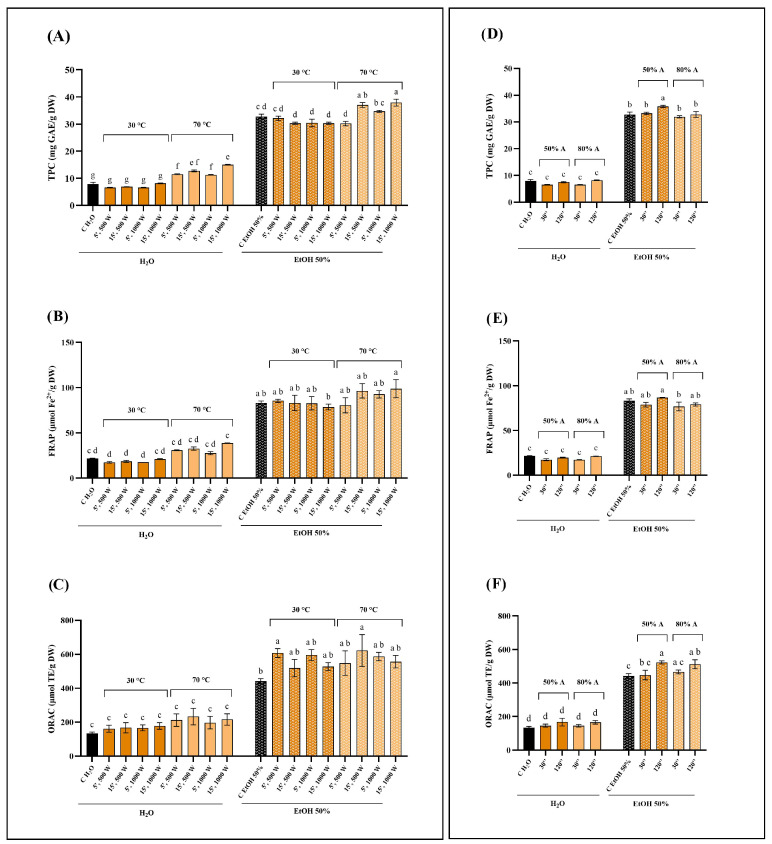
Comparison of total phenolic content (TPC; panels (**A**,**D**)), ferric reducing antioxidant power (FRAP; panels (**B**,**E**)), and oxygen reducing antioxidant capacity (ORAC; panels (**C**,**F**)) of water and 50% ethanol Norway spruce bark (NSB) extracts from conventional extraction (C), microwave-assisted extraction (MAE; panels (**A**–**C**)) and ultrasound-assisted extraction (UAE; panels (**D**–**F**)). Data were analyzed by one-way ANOVA followed by Tukey’s post hoc test. Different letters indicate statistically significant differences among treatments (*p* < 0.05). DW: dry weight; GAE: gallic acid equivalents; EtOH: ethanol; A: amplitude.

**Figure 2:**
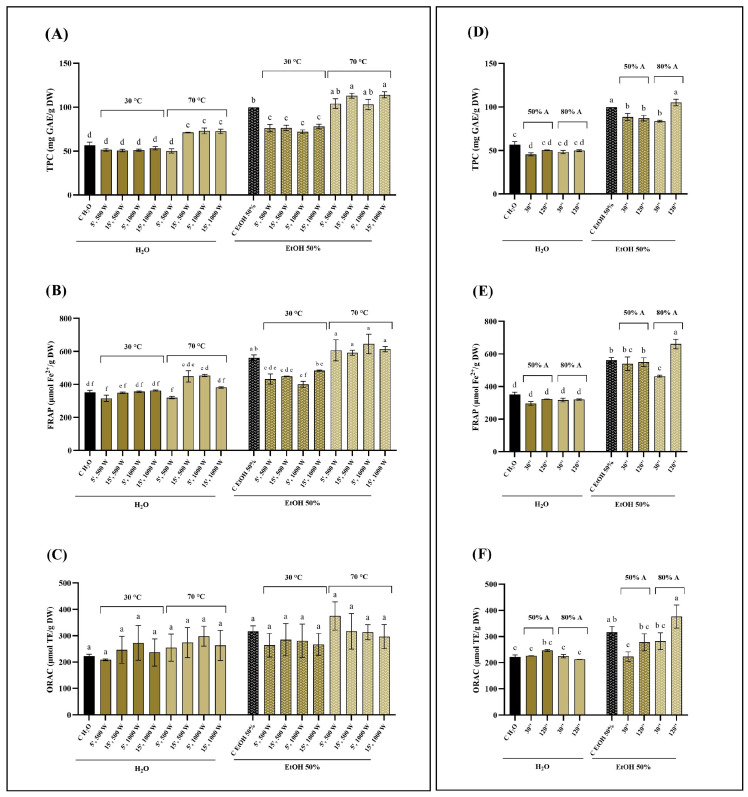
Comparison of total phenolic content (TPC; panels (**A**,**D**)), ferric reducing antioxidant power (FRAP; panels (**B**,**E**)), and oxygen reducing antioxidant capacity (ORAC; panels (**C**,**F**)) of water and 50% ethanol chestnut tree wood (CTW) extracts from conventional extraction (C), microwave-assisted extraction (MAE; panels (**A**–**C**)) and ultrasound-assisted extraction (UAE; panels (**D**–**F**)). Data were analyzed by one-way ANOVA followed by Tukey’s post hoc test. Different letters indicate statistically significant differences among treatments (*p* < 0.05). DW: dry weight; GAE: gallic acid equivalents; EtOH: ethanol; A: amplitude.

**Figure 3:**
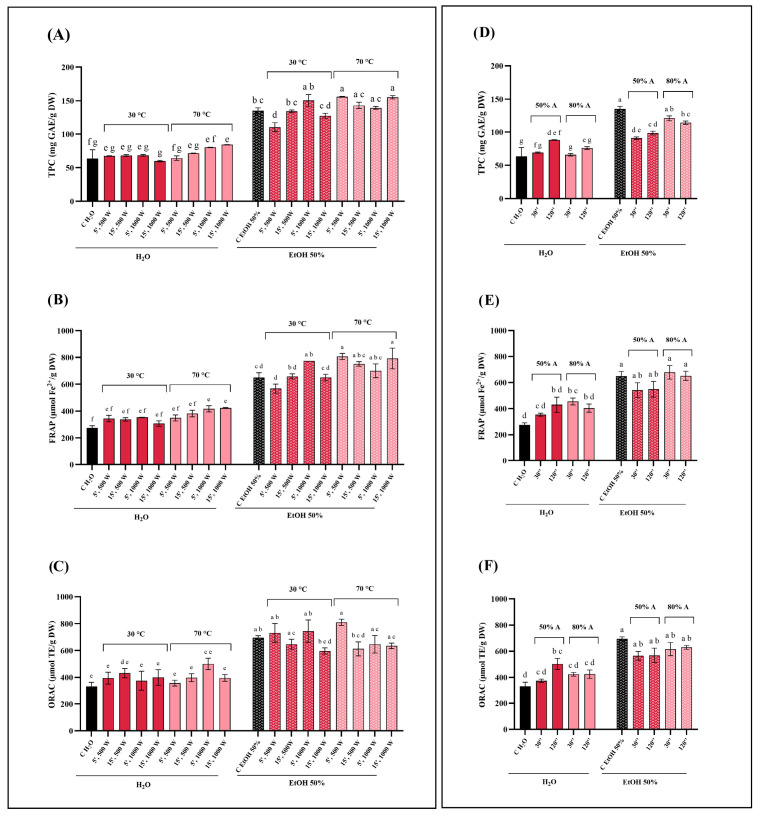
Comparison of total phenolic content (TPC; panels (**A**,**D**)), ferric reducing antioxidant power (FRAP; panels (**B**,**E**)), and oxygen reducing antioxidant capacity (ORAC; panels (**C**,**F**)) of water and 50% ethanol pomegranate fruit waste (PFW) extracts from conventional extraction (C), microwave-assisted extraction (MAE; panels (**A**–**C**)) and ultrasound-assisted extraction (UAE; panels (**D**–**F**)). Data were analyzed by one-way ANOVA followed by Tukey’s post hoc test. Different letters indicate statistically significant differences among treatments (*p* < 0.05). DW: dry weight; GAE: gallic acid equivalents; EtOH: ethanol; A: amplitude.

The results obtained for Norway spruce bark (NSB), chestnut tree wood (CTW), and pomegranate fruit waste (PFW) are reported in Figure 1, Figure 2, and Figure 3, respectively. Overall, the extraction solvent emerged as the most influential parameter across all matrices. The most pronounced solvent-dependent differences were observed for NSB: both MAE and UAE performed with 50% ethanol consistently resulted in significantly higher extraction efficiencies than water, yielding up to three-fold higher TPC values (Figure 1A,D). In contrast, CTW exhibited more moderate increases when ethanol was used (Figure 2). Regarding PFW, hydroalcoholic extraction with MAE nearly doubled the phenolic recovery compared to aqueous extraction (Figure 3A). These results are consistent with several studies reporting that, across multiple matrices (e.g., peel, pomace, bark, and rhizomes), hydroalcoholic mixtures (50–60%) represent the solvent of choice for phenolic recovery in MAE and UAE [27,28,29,30]. Indeed, the increase in polarity achieved by adding water to the organic solvent improves microwave energy absorption and enhances temperature development, thereby improving extraction efficiency [31].

Regarding MAE, extraction temperature had a substantial impact on phenolic recovery and antioxidant activity. This effect was particularly evident for the CTW matrix, where extraction at 70 °C resulted in significantly higher TPC and FRAP values compared to 30 °C, while ORAC appeared less sensitive to these conditions (Figure 2). In NSB extracts, higher temperatures in MAE enhanced TPC values, especially when combined with longer extraction times (15 min) (Figure 1A). Conversely, the effect of temperature was less pronounced for PFW, although significant increases at 70 °C were still detected, particularly in TPC and FRAP assays (Figure 3A,B). Temperature optimization is widely recognized as a critical factor in phenolic compounds recovery. In general, increasing temperature improves extraction efficiency; however, exceeding matrix-specific thresholds may result in compound degradation, as reported in several works [32,33,34].

Microwave power alone did not exert a consistent influence on extraction efficiency across the tested matrices, as no clear or systematic trends were observed when other parameters were kept constant. In contrast, extending extraction time positively influenced phenolic recovery in selected cases, most notably for NSB and only in hydroalcoholic extracts (Figure 1A). However, for PFW, in some cases, the shorter extraction time (5 min) resulted in higher phenolic recovery, suggesting a possible onset of compound degradation or saturation effects (Figure 3A). This non-monotonous behavior of microwave power and extraction time has been previously encountered in the literature. For example, Hong and colleagues [35] found that neither variable had a significant effect on total phenolic content or overall extraction yield from grape seed when solvent composition was kept constant. Similarly, in a study on *Scutellaria* spp., increasing microwave power and extraction times did not consistently increase TPC; conversely, higher power levels even reduced phenolic content in some cases, suggesting degradation at excessive power [36].

Variations in ultrasound amplitude produced only limited effects overall, although matrix-dependent responses were observed. For instance, increasing the amplitude to 80% slightly enhanced TPC and antioxidant activity in hydroalcoholic PFW extracts, albeit not always significantly (Figure 3D). In CTW extracts, however, the combination of 80% amplitude, 120 s extraction time, and 50% ethanol resulted in significantly higher phenolic recovery than the same conditions at 60% amplitude (Figure 2D). These findings align with previous UAE studies indicating that ultrasound amplitude alone often has a limited or non-linear influence on phenolic extraction; on the other hand, matrix- and solvent-specific interactions may enhance TPC and antioxidant activity when higher amplitudes are combined with appropriate extraction times. For instance, in the case of *Centella asiatica*, ultrasonic power modulation had no significant effects compared to other parameters [37]. Instead, in lime by-products, increasing ultrasound amplitude led to higher TPC levels, although the effect depended strongly on the extraction conditions [38]. These observations stress the importance of structural and compositional differences among biomass types. Lignocellulosic matrices such as bark and wood are characterized by rigid cell walls and complex interactions between phenolics and structural polymers (e.g., lignin and cellulose), which may require more intense disruption mechanisms, such as microwave heating. In contrast, softer plant tissues or fruit-derived matrices may respond differently to cavitation effects during UAE. These differences may explain why ultrasound amplitude enhancements reported for other matrices are not universally observed, as extraction efficiency depends on the interplay between matrix architecture and process conditions [39,40,41]. Overall, both MAE and UAE yielded phenolic content and antioxidant activities that were at least comparable to, and in several cases higher than, those obtained by conventional 2 h maceration at room temperature. The dominant role of solvent composition observed across all matrices can be attributed to its effect on both polarity and mass transfer phenomena, influencing the solubility and release of phenolic compounds [42,43]. Although no formal multivariate experimental design was applied, the consistency of these trends across independent assays supports the conclusion that solvent selection represents the primary determinant of extraction efficiency under the tested conditions.

**Figure 4:**
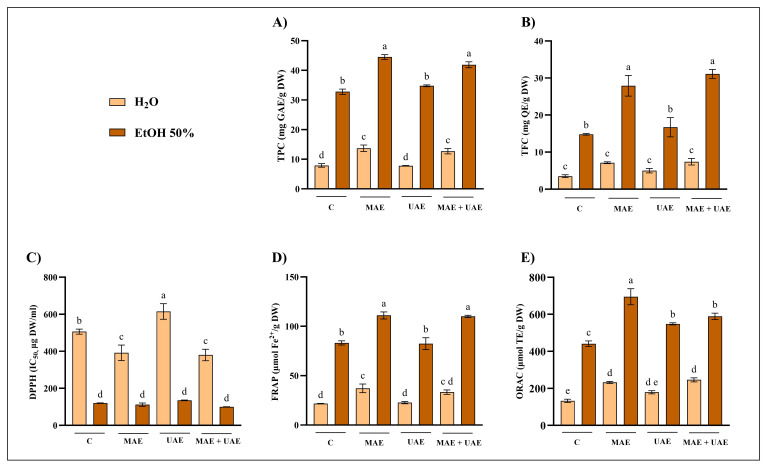
Optimized extraction conditions for NSB. Comparison of (**A**) total phenolic content (TPC), (**B**) total flavonoid content (TFC), (**C**) 2-diphenyl-1-picrylhydrazyl (DPPH), (**D**) ferric reducing antioxidant power (FRAP), and (**E**) oxygen radical absorbance capacity (ORAC) assays of Norway spruce bark (NSB) extracts from conventional extraction (C), microwave-assisted extraction (MAE; H_2_O: 70 °C, 1000 W, 15 min; EtOH 50%: 70 °C, 500 W, 15 min), ultrasound-assisted extraction (UAE; H_2_O: 80% A, 120 s; EtOH 50%: 50% A, 120 s) and their combination (MAE + UAE). Data were analyzed by one-way ANOVA followed by Tukey’s post hoc test. Different letters indicate statistically significant differences among treatments (*p* < 0.05). DW: dry weight; EtOH: ethanol; IC: inhibitory concentration; GAE: gallic acid equivalents; QE: quercetin equivalents; TE: Trolox equivalents.

**Figure 5:**
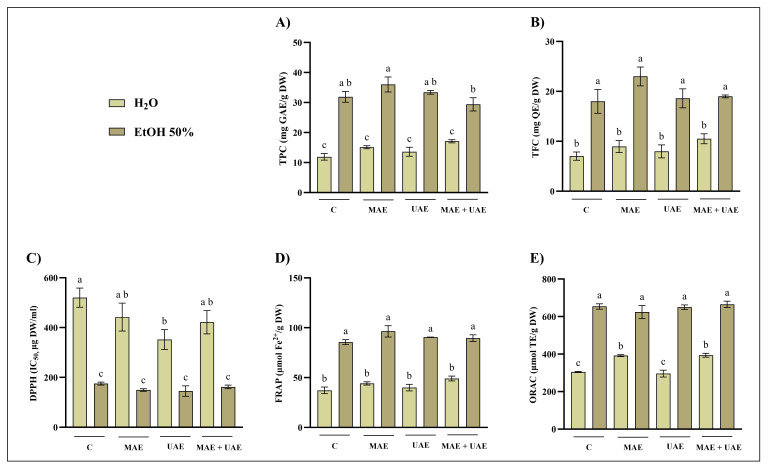
Optimized extraction conditions for NST. Comparison of (**A**) total phenolic content (TPC), (**B**) total flavonoid content (TFC), (**C**) 2-diphenyl-1-picrylhydrazyl (DPPH), (**D**) ferric reducing antioxidant power (FRAP), and (**E**) oxygen radical absorbance capacity (ORAC) assays of Norway spruce twigs (NST) extracts from conventional extraction (C), microwave-assisted extraction (MAE; H_2_O: 70 °C, 1000 W, 15 min; EtOH 50%: 70 °C, 500 W, 15 min), ultrasound-assisted extraction (UAE; H_2_O: 80% A, 120 s; EtOH 50%: 50% A, 120 s) and their combination (MAE + UAE). Data were analyzed by one-way ANOVA followed by Tukey’s post hoc test. Different letters indicate statistically significant differences among treatments (*p* < 0.05). DW: dry weight; EtOH: ethanol; IC: inhibitory concentration; GAE: gallic acid equivalents; QE: quercetin equivalents; TE: Trolox equivalents.

**Figure 6:**
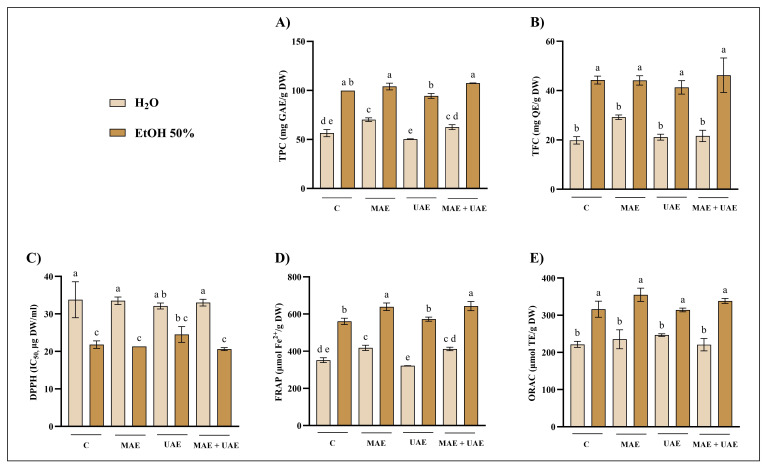
Optimized extraction conditions for CTW. Comparison of (**A**) total phenolic content (TPC), (**B**) total flavonoid content (TFC), (**C**) 2-diphenyl-1-picrylhydrazyl (DPPH), (**D**) ferric reducing antioxidant power (FRAP), and (**E**) oxygen radical absorbance capacity (ORAC) assays of chestnut tree wood (CTW) extracts from conventional extraction (C), microwave-assisted extraction (MAE; H_2_O: 70 °C, 1000 W, 15 min; EtOH 50%: 70 °C, 500 W, 15 min), ultrasound-assisted extraction (UAE; H_2_O: 80% A, 120 s; EtOH 50%: 50% A, 120 s) and their combination (MAE + UAE). Data were analyzed by one-way ANOVA followed by Tukey’s post hoc test. Different letters indicate statistically significant differences among treatments (*p* < 0.05). DW: dry weight; EtOH: ethanol; IC: inhibitory concentration; GAE: gallic acid equivalents; QE: quercetin equivalents; TE: Trolox equivalents.

**Figure 7:**
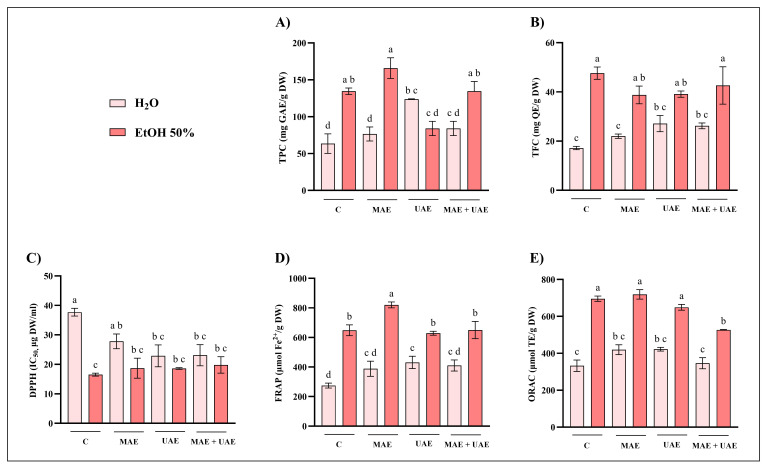
Optimized extraction conditions for PFW. Comparison of (**A**) total phenolic content (TPC), (**B**) total flavonoid content (TFC), (**C**) 2-diphenyl-1-picrylhydrazyl (DPPH), (**D**) ferric reducing antioxidant power (FRAP), and (**E**) oxygen reducing antioxidant capacity (ORAC) assays of pomegranate fruit waste (PFW) extracts from conventional extraction (C), microwave-assisted extraction (MAE; H_2_O: 70 °C, 1000 W, 15 min; EtOH 50%: 70 °C, 500 W, 15 min), ultrasound-assisted extraction (UAE; H_2_O: 80% A, 120 s; EtOH 50%: 50% A, 120 s) and their combination (MAE + UAE). Data were analyzed by one-way ANOVA followed by Tukey’s post hoc test. Different letters indicate statistically significant differences among treatments (*p* < 0.05). DW: dry weight; EtOH: ethanol; IC: inhibitory concentration; GAE: gallic acid equivalents; QE: quercetin equivalents; TE: Trolox equivalents.

The optimal combinations of time and microwave power or ultrasound amplitude, for each solvent and plant material, were selected based on comparison with the conventional extraction method. The selection criteria prioritized conditions yielding the highest recovery in at least one assay. Where differences were not statistically significant, shorter processing times or lower energy inputs were preferred, in line with green extraction principles. Accordingly, NST extracts were prepared using the optimal parameters identified for NSB to ensure methodological consistency across similar matrices. Furthermore, for the selected extraction conditions, additional assays (total flavonoid content, TFC, and DPPH radical scavenging activity) were performed to further validate the results, and a combined MAE–UAE approach was also investigated (Figure 4, Figure 5, Figure 6 and Figure 7).

Several consistent and matrix-dependent trends were observed. Overall, higher levels of total phenolics and flavonoids were closely associated with stronger antiradical and antioxidant activities in DPPH, FRAP, and ORAC assays. This trend is well documented, as phenolic compounds are recognized as major contributors to antioxidant capacity due to their redox properties and ability to scavenge free radicals [44].

In the present study, MAE outperformed UAE in terms of phenolic recovery and antioxidant activity across all matrices (Figure 4, Figure 5, Figure 6 and Figure 7). This result may partly be attributed to the shorter UAE extraction time, imposed by rapid solvent heating. This observation agrees with several comparative studies reporting higher yields and efficiency for MAE relative to UAE [45,46,47].

Additionally, the combined application of UAE + MAE did not generally result in statistically significant improvements in phenolic recovery compared to MAE alone. In most matrices, UAE + MAE yields were higher than UAE but comparable to MAE, suggesting that ultrasound does not provide additional benefits when microwave-induced cell disruption is already effective (Figure 4, Figure 5, Figure 6 and Figure 7).

This observation is consistent with reports indicating no additive effects of combined techniques once MAE is optimized [48]. Among the four matrices, PFW exhibited the highest content of total phenolics and antioxidant and radical scavenging activities (Figure 7), followed by CTW (Figure 6). Notably, CTW showed particularly strong activity in DPPH (Figure 6C) and FRAP (Figure 6D) assays but not in ORAC (Figure 6E), in contrast to NSB and NST. This difference likely reflects variations in phenolic composition, as extracts rich in hydrolysable tannins typically exhibit strong electron-transfer mechanisms relevant to DPPH and FRAP assays. Taken together, these data supported the selection of one optimal extract per waste material for advanced characterization and biological evaluation:

• NSB: 50% EtOH, 70 °C, 500 W, 15 min
• NST: 50% EtOH, 70 °C, 500 W, 15 min
• CTW: 50% EtOH, 70 °C, 500 W, 15 min
• PFW: 50% EtOH, 70 °C, 500 W, 5 min

These conditions provide an effective balance between maximizing extraction yield and minimizing processing time or energy consumption, in accordance with sustainable extraction principles.

### 3.2. Stability of the Extracts

Extract stability represents a key parameter for evaluating their potential industrial applicability. The stability of the optimized extracts was assessed three weeks after preparation by monitoring TPC levels (Table 2).

Except for CTW, all extracts showed a decrease in TPC over time, which is consistent with the well-documented instability of phenolic compounds in solutions. Phenolics may undergo oxidative degradation, polymerization, and structural rearrangements during storage, particularly in aqueous or hydroalcoholic systems [49]. Despite these changes, all extracts retained relatively high phenolic levels after storage, indicating overall good stability. PFW extract exhibited the greatest reduction, suggesting a higher susceptibility of its phenolic profile to degradation processes. The increase observed for CTW extracts may not reflect a true increase in total phenolic content, but rather the hydrolysis of complex tannins into smaller, more reactive phenolic compounds that are more readily detected by the Folin–Ciocalteu assay. This phenomenon has been previously reported for tannin-rich matrices and highlights the limitations of spectrophotometric assays in distinguishing between structural transformations and absolute concentration changes [50,51]. A detailed kinetic analysis of phenolic degradation was beyond the scope of this study and should be addressed in future investigations. Overall, these findings suggest that the stability of phenolic compounds is strongly influenced by extract composition and solvent system, highlighting the importance of optimizing storage conditions to preserve bioactive properties.

### 3.3. HPLC-DAD Profiling

**Figure 8:**
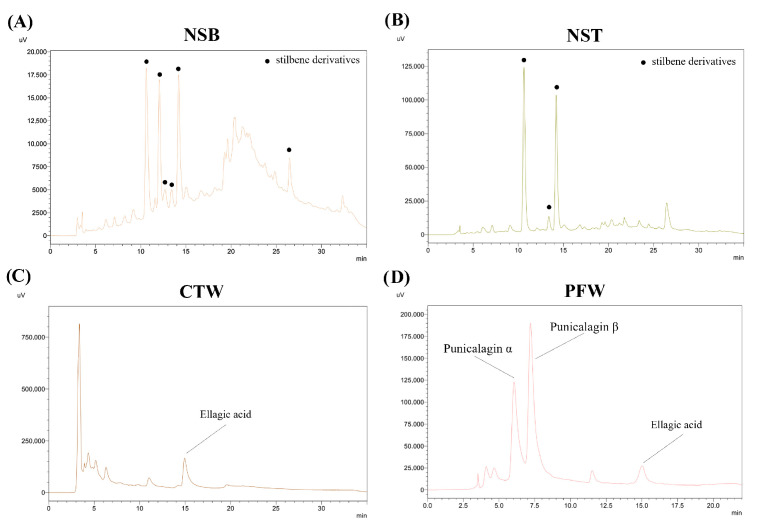
Chromatograms of optimized extracts. (**A**) Norway spruce bark (NSB), (**B**) Norway spruce twigs (NST), (**C**) chestnut tree wood (CTW), and (**D**) pomegranate fruit waste (PFW), acquired at 330 nm (NSB, NST), 254 nm (CTW), and 375 nm (PFW). The main peaks corresponding to the identified compounds are labeled in the chromatograms.

After optimization, all selected extracts underwent HPLC-DAD analysis to quantify the main phenolic compounds (Figure 8, Table 3). Stilbene derivatives, quantified as resveratrol equivalents, were the major peaks detected in both Norway spruce bark and twig extracts, with the latter exhibiting approximately a four-fold higher level despite similar antioxidant activities (Figure 8A,B; Table 3). They are a class of structurally simple phenolics, with an ethylene link connecting two hydroxylated benzene rings, known to act as phytoalexins in plant tissues, thereby constituting both a defense towards the attack of detrimental microorganisms to the plant tissue, and a valuable source of antimicrobial agents for the human being [52]. Furthermore, the medicinal value of stilbenes has been recognized, especially thanks to the beneficial effects they exert on cardiovascular diseases, extensively studied in clinical trials [53]. Our findings align with previous reports of stilbenes in conifer bark residues, where they co-occur with a complex mixture of phenolics. Indeed, several stilbenes and other simple phenolics were isolated from the root bark of Norway spruce [54]. Additionally, Piyaratne et al. [55] reported that spruce bark contains considerable amounts of resveratrol, highlighting conifer bark residues as potential stilbene sources, although total phenolic content may not directly correlate with resveratrol levels.

Chestnut tree wood extract and pomegranate by-products were dominated by ellagic acid and ellagitannins. Ellagitannins are complex plant polyphenols composed of hexahydroxydiphenoyl moieties esterified to a monosaccharide, and they occur in several berries and seeds. The abundance of aromatic hydroxyl groups guarantees enhanced reactivity in biological systems, where their antioxidant, antimicrobial, anti-inflammatory, and anticancer effects have been recognized [56].

CTW displayed a prominent ellagic acid peak (Figure 8C), consistent with the well-documented abundance of ellagitannins in chestnut tissues, whose hydrolysis yields free ellagic acid. Reported ellagic acid concentrations in chestnut bark and related tissues vary widely (<1 to ~20 mg/g in unhydrolyzed extracts), with extraction efficiency strongly dependent on solvent and method, highlighting the importance of process optimization for phenolic recovery [57,58].

Punicalagin isomers α and β dominated PFW, while ellagic acid was also present in a similar amount compared to CTW (Figure 8D). The punicalagin content in our extract falls within the ranges reported in previous studies, considering that the starting material consisted of a mixture of peels, seeds, and pulp. Since punicalagins are highly concentrated in the peel but present at much lower levels in seeds and pulp, the overall content in mixed waste reflects this distribution [59]. Compared to conventional methods, MAE has been shown to enhance the recovery of punicalagins and other phenolics from fruit wastes, supporting the combined use of MAE/UAE approaches for the valorization of these matrices [60].

The presence of these compounds supports the functional potential of the extracts investigated. However, compound-specific stability during storage was not assessed, and changes in chromatographic profiles over time may provide further insight and should be addressed in future studies

### 3.4. Modulation of Carbohydrate- and Lipid-Metabolizing Enzymes by Optimized Extracts

The best-performing extracts selected during the optimization process were evaluated for their ability to modulate the activity of enzymes involved in carbohydrate metabolism (α-amylase and DPP-IV) and lipid metabolism (pancreatic lipase), aiming to assess their potential antidiabetic and anti-obesity properties.

Overall, none of the tested extracts exhibited significant inhibitory activity against DPP-IV (IC_50_ of diprotin A = 8.11 ± 1.0 μg/mL) or pancreatic lipase (% inhibition by Orlistat = 45.1 ± 7.1 at 0.02 mM). These results are consistent with previous studies showing that many plant extracts rich in polyphenols exhibit limited inhibitory activity against DPP-IV unless they are highly concentrated or specifically enriched in active molecules such as flavonoids, phenolic acids, or peptides, with reported in vitro inhibition often in the high μg/mL range [61]. Similarly, the literature on polyphenol-mediated pancreatic lipase inhibition indicates that only specific flavonoids and phenolic acids exert measurable effects on lipase activity through hydrogen bonding and hydrophobic interactions, whereas crude extracts often show modest inhibition at comparable concentrations [62].

In contrast, the extracts were more effective against α-amylase, an enzyme involved in starch hydrolysis and a key target for controlling postprandial hyperglycemia. Extracts were tested at final concentrations ranging from 43.5 to 348.0 μg/mL. While NST and CTW showed no significant effect, PFW and NSB inhibited α-amylase activity with IC_50_ values of 584.0 ± 20.4 μg/mL and 114.9 ± 33.8 μg/mL, respectively. For comparison, the reference inhibitor acarbose exhibited 99.3 ± 0.97% inhibition at 92.6 μg/mL.

These results suggest that the observed α-amylase inhibition correlates with the content of polyphenolic compounds, particularly ellagitannins in PFW and stilbenes in NSB, which have been previously reported to interfere with carbohydrate-hydrolyzing enzymes [63,64]. Interestingly, NST did not show α-amylase inhibition despite its higher stilbenoid content. One possible explanation is that, in addition to stilbenes, Norway spruce bark contains a significant fraction of condensed tannins (proanthocyanidins), primarily procyanidins composed of flavan-3-ol units. These oligomeric tannins can form multiple hydrogen bonds and hydrophobic interactions with enzyme active sites, potentially contributing to enzyme inhibition more effectively than stilbenoid monomers alone, as reported in the previous chemical characterization of spruce bark extracts [65,66]. This observation could also help explain the four-fold higher inhibitory activity of NSB over PFW: the former is characterized by procyanidins, whereas the latter by ellagitannins, which may exert different and class-specific α-amylase inhibition mechanisms. Despite the substantially lower activity compared to acarbose, these findings indicate that NSB and PFW, as lignocellulosic and fruit waste extracts, possess inhibitory activity against α-amylase, supporting their potential application in functional foods targeting postprandial glycemic control. These results suggest that enzyme inhibition is not only dependent on total phenolic content but also on the specific chemical structure and interactions of individual compounds with enzyme active sites. Further studies, including mechanistic assays and in vivo evaluations, are necessary to confirm their antidiabetic potential and elucidate their structure–activity relationships.

### 3.5. Antimicrobial Activity of Optimized Extracts

**Figure 9:**
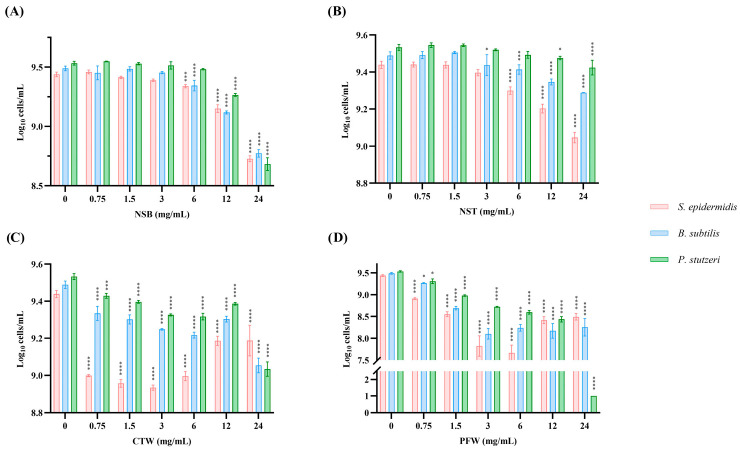
Antimicrobial activity of the optimized extracts. (**A**) Norway spruce bark (NSB), (**B**) Norway spruce twigs (NST), (**C**) chestnut tree wood (CTW), (**D**) pomegranate fruit waste (PFW) against the selected bacterial strains (*Staphylococcus epidermidis*, *Bacillus subtilis*, and *Pseudomonas stutzeri*). Two-way ANOVA, Dunnett’s post hoc test. * *p* < 0.05; *** *p* < 0.001; **** *p* < 0.0001.

The antimicrobial efficacy of the best-performing extracts was evaluated against three bacterial strains selected as representative microbial targets in cosmetics, food, and environmental contexts. *Staphylococcus epidermidis* (Gram-positive) is a common commensal of human skin, but it can act as an opportunistic pathogen, contributing to skin infections, biofilm formation, and product contamination in cosmetic formulations [67]. *Bacillus subtilis* is a Gram-positive bacterium frequently associated with food spoilage and reduced shelf life, making it a relevant indicator organism for evaluating antimicrobial strategies in food preservation [68]. Instead, *Pseudomonas stutzeri* (Gram-negative) is an environmental bacterium widely distributed in soil and water systems, generally considered non-pathogenic, but representing a good model for assessing extract efficacy against resilient Gram-negative bacteria of environmental and industrial relevance [69]. Bacterial growth was monitored as log_10_ cells/mL after exposure to increasing extract concentrations ranging from 0 to 24 mg/mL (Figure 9).

Both NSB and NST demonstrated a clear dose-dependent antimicrobial effect across all tested strains, with a pronounced reduction in estimated cell counts observed at the highest concentrations (12 and 24 mg/mL) (Figure 9A,B). Among the three microbial strains, *S. epidermidis* was the most susceptible to NST, followed by *B. subtilis* and *P. stutzeri*. In contrast, NSB exerted a more uniform inhibitory effect across all microorganisms. Overall, NSB achieved slightly higher inhibition than NST, consistent with previous observations on enzymatic activity and suggesting differences in phytochemical composition. In particular, the bark extract may contain higher concentrations or more potent antimicrobial constituents compared to the twig extract. Previous studies have reported antimicrobial activity of spruce bark extracts against both Gram-positive and Gram-negative bacteria, supporting their potential as antimicrobial agents [70]. Comparative analyses of conifer needle and bark extracts, including spruce, have also demonstrated variable antimicrobial potency across extracts, consistent with differences in phytochemical composition [71].

CTW exhibited a more selective antimicrobial profile, showing preferential inhibition of *S. epidermidis* at lower concentrations (0.75 mg/mL) (Figure 9C). However, a clear dose–response relationship was not observed, and the inhibitory effect appeared less pronounced at higher concentrations (12 and 24 mg/mL), which may indicate a threshold-dependent activity or the presence of compounds exerting antagonistic or saturating effects at higher concentrations. Extracts of chestnut wood and other chestnut byproducts rich in tannins and other phenolics have demonstrated significant in vitro antimicrobial activity against both Gram-positive and Gram-negative bacteria, with inhibitory concentrations comparable to standard antibiotics at higher doses [72].

PFW displayed a distinct and highly effective antimicrobial profile (Figure 9D). It exerted a significant inhibitory effect against the Gram-negative *P. stutzeri* at all tested concentrations, with a drastic reduction in bacterial growth at 24 mg/mL where log_10_ cells/mL values approached near-eradication levels. Activity against *B. subtilis* and *S. epidermidis* was comparatively less pronounced but remained evident and stronger than that observed for the other extracts. This behavior is consistent with the high content of punicalagins and other hydrolysable tannins in pomegranate by-products, which have been widely reported to possess broad-spectrum antimicrobial activity [73,74].

Among the tested extracts, PFW emerged as the most potent antimicrobial agent, while NSB showed balanced broad-spectrum activity. Overall, these data suggest that extracts rich in hydrolysable or condensed tannins (PFW, NSB, and CTW) exhibit consistent antimicrobial activity [75]. The results support the potential use of these plant-derived waste extracts as natural antimicrobial agents in food preservation, cosmetics, or topical formulations targeting skin-associated bacteria. Further studies are warranted to identify the specific active compounds and elucidate their mechanisms of action.

## 4. Conclusions

This study highlights the potential of agri-food and forestry by-products as sustainable sources of phenolic compounds with relevant biological activities. The use of green extraction techniques, particularly MAE, significantly improved extraction efficiency while reducing processing time compared with conventional methods. Among the investigated matrices, pomegranate waste and chestnut wood extracts exhibited the highest phenolic contents and antioxidant capacities, confirming their suitability as functional ingredients. Phytochemical profiling revealed the presence of key bioactive compounds, including stilbenes, ellagic acid, and punicalagins, known for their antioxidant and antimicrobial properties. Biological assays demonstrated moderate enzyme inhibitory activity and promising antimicrobial effects, supporting the potential use of these extracts as natural additives or preservative agents. Although total phenolic content changed during storage (decreasing in spruce and pomegranate extracts but increasing in chestnut wood), the extracts generally maintained high phenolic content, indicating acceptable short-term stability for potential applications. These findings suggest that plant-derived by-products can be efficiently valorized through sustainable extraction processes and used in the formulation of functional foods, nutraceuticals, cosmetic ingredients, and natural antimicrobial systems. While the results demonstrate promising biofunctional properties, the present study represents a preliminary assessment of extract stability and biological activity. Therefore, further investigations are required to evaluate long-term stability, compound-specific behavior, and efficacy in real systems. Future work should also address process scale-up and formulation aspects to support potential industrial applications.

## Figures and Tables

**Table 1:** Parameters selected for comparison between conventional, microwave-assisted extraction (MAE), and ultrasound-assisted extraction (UAE).

	Solvent	Temperature	Time
**Conventional**	Water, 50% EtOH	Room temperature	2 h
**MAE**		30 °C, 70 °C	5 min, 15 min
**UAE**			30 s, 120 s

**Table 2:** Stability of total phenolic content (mg GAE/g dw) monitored over 21 days of storage of optimized extracts from Norway spruce bark (NSB), Norway spruce twigs (NST), chestnut tree wood (CTW), and pomegranate fruit waste (PFW). Paired Student’s *t*-test. Significance levels are indicated as follows: * *p* < 0.05; ** *p* < 0.01; *** *p* < 0.001. *n* = 4.

	Day 1	Day 21	% Change
**NSB**	44.5 ± 0.8	37.0 ± 0.5 ***	−16.8
**NST**	36.0 ± 2.5	29.7 ± 2.7 *	−17.5
**CTW**	104.0 ± 3.4	118.0 ± 1.6 **	+13.5
**PFW**	165.8 ± 14.1	124.3 ± 6.8 **	−25.0

**Table 3:** Quantification of specific compounds and classes of compounds in the optimized extracts from Norway spruce bark (NSB), Norway spruce twigs (NST), chestnut tree wood (CTW), and pomegranate fruit waste (PFW).

Extract	Standard Compound	Wavelength (nm)	Quantification (mg/g dw)	Total Chromatogram Area
**NSB**	Resveratrol	330	0.57 ± 0.069 ^a^	336.6 ± 30.6
**NST**			2.10 ± 0.23 ^a^	883.1 ± 41.9
**CTW**	Ellagic acid	254	4.21 ± 0.077	5535.2 ± 77.9
**PFW**	Punicalagins (α + β)	375	52.2 ± 4.0	1358.7 ± 93.8
	Ellagic acid	254	2.49 ± 0.098	897.2 ± 87.8

^a^ stilbene derivatives, expressed as resveratrol equivalents.

## Data Availability

The original contributions presented in this study are included in the article/Supplementary Material. Further inquiries can be directed to the corresponding author.

## Supplementary Materials

The following supporting information can be downloaded at: https://www.mdpi.com/article/10.3390/foods15081422/s1, Figure S1: Schematic representation of the extraction workflow and instrumentation used. NSB: Norway spruce bark; NST: Norway spruce twigs; CTW: chestnut tree wood; PFW: pomegranate fruit waste (PFW); MAE: microwave-assisted extraction; UAE: ultrasound-assisted extraction.
