# Supplementary material for: Sustainable Recovery and Biofunctional Characterization of Polyphenol-Rich Extracts from Norway Spruce, Chestnut Wood, and Pomegranate By-Products
Source: Foods. 2026 Apr 19;15(8):1422. doi: 10.3390/foods15081422 (PMC13115244; doi:10.3390/foods15081422)
Supplement: Supplementary file 1 [file foods-15-01422-s001.zip › foods-4227986-supplementary.pdf]

Supplementary material

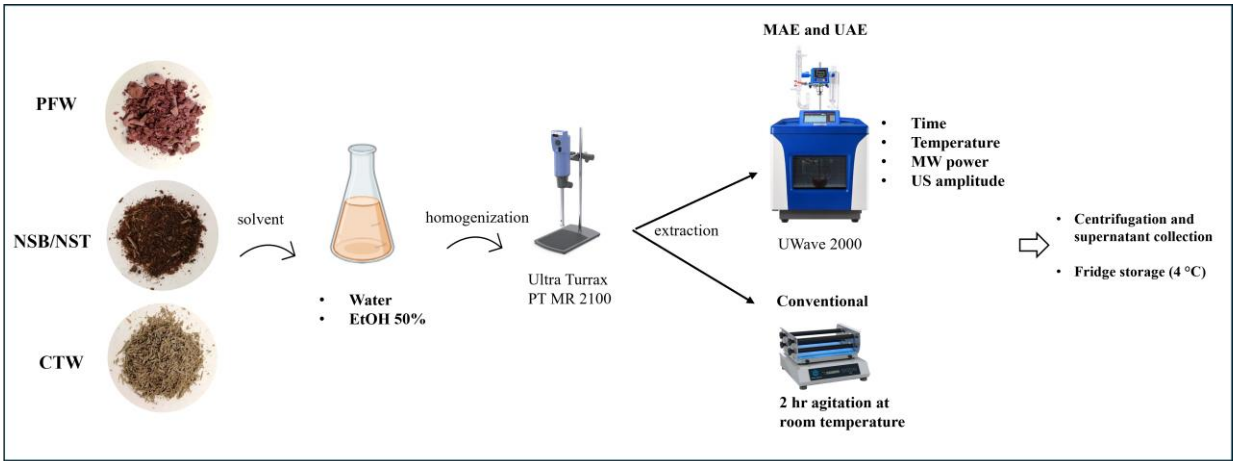

Figure S1. Schematic representation of the extraction workflow and instrumentation used. NSB: Norway spruce bark; NST: Norway spruce twigs; CTW: chestnut tree wood; PFW: pomegranate fruit waste (PFW); MAE: microwave-assisted extraction; UAE: ultrasound-assisted extraction.
